# Foods with Potential Prooxidant and Antioxidant Effects Involved in Parkinson's Disease

**DOI:** 10.1155/2020/6281454

**Published:** 2020-08-03

**Authors:** Alejandra Guillermina Miranda-Díaz, Andrés García-Sánchez, Ernesto Germán Cardona-Muñoz

**Affiliations:** Department of Physiology, University Health Sciences Center, University of Guadalajara, Guadalajara, Jalisco, Mexico

## Abstract

Oxidative stress plays a fundamental role in the pathogenesis of Parkinson's disease (PD). Oxidative stress appears to be responsible for the gradual dysfunction that manifests via numerous cellular pathways throughout PD progression. This review will describe the prooxidant effect of excessive consumption of processed food. Processed meat can affect health due to its high sodium content, advanced lipid oxidation end-products, cholesterol, and free fatty acids. During cooking, lipids can react with proteins to form advanced end-products of lipid oxidation. Excessive consumption of different types of carbohydrates is a risk factor for PD. The antioxidant effects of some foods in the regular diet provide an inconclusive interpretation of the environment's mechanisms with the modulation of oxidation stress-induced PD. Some antioxidant molecules are known whose primary mechanism is the neuroprotective effect. The melatonin mechanism consists of neutralizing reactive oxygen species (ROS) and inducing antioxidant enzyme's expression and activity. N-acetylcysteine protects against the development of PD by restoring levels of brain glutathione. The balanced administration of vitamin B3, ascorbic acid, vitamin D and the intake of caffeine every day seem beneficial for brain health in PD. Excessive chocolate intake could have adverse effects in PD patients. The findings reported to date do not provide clear benefits for a possible efficient therapeutic intervention by consuming the nutrients that are consumed regularly.

## 1. Introduction

Parkinson's disease (PD) is the second most common chronic progressive neurodegenerative disorder. PD is characterized by the selective loss of dopaminergic neurons of the substantia nigra (SN) pars compacta, which conditions deficiency of dopamine secretion in the basal ganglia of the midbrain with the ability to produce classic motor symptoms: bradykinesia, tremor, rigidity, posterior postural instability, gait disturbances, smell, memory, and dementia [1]. PD involves genetic, environmental, and toxicological factors [2, 3]. PD is associated with oxide-reduction processes through excessive production of reactive oxygen spices (ROS) [4]. The hallmark of PD is the appearance of insoluble inclusions in neurons called Lewy bodies. Lewy bodies mainly consist of *α*-synuclein deposition [5]. *α*-Synuclein is a 140 kDa protein encoded via the SNCA gene. *α*-Synuclein plays an essential role in the pathogenesis of PD. Duplication, triplication, and point mutations in the N-terminal region (A30P, A53T, and E46K) are linked to familial PD [6]. A recent study suggests that *α*-synuclein monomers and tetramers are the physiological forms, while oligomers and fibrils are the pathogenic forms [7]. Abnormal accumulation of soluble *α*-synuclein monomers may lead to the formation of oligomers and fibrils as a key pathogenic event in the early stages of PD [8]. The first clinical signs and symptoms of PD appear after the loss of 50-70% of SN [9, 10]. Based on PD's progressive nature, oxidation might be responsible for gradual dysfunction as a continuous process that manifests itself through many cellular pathways throughout the disease. ROS are normally produced in the cell during the mitochondrial electron transfer chain or by redox reactions [11].

**Figure 1:**
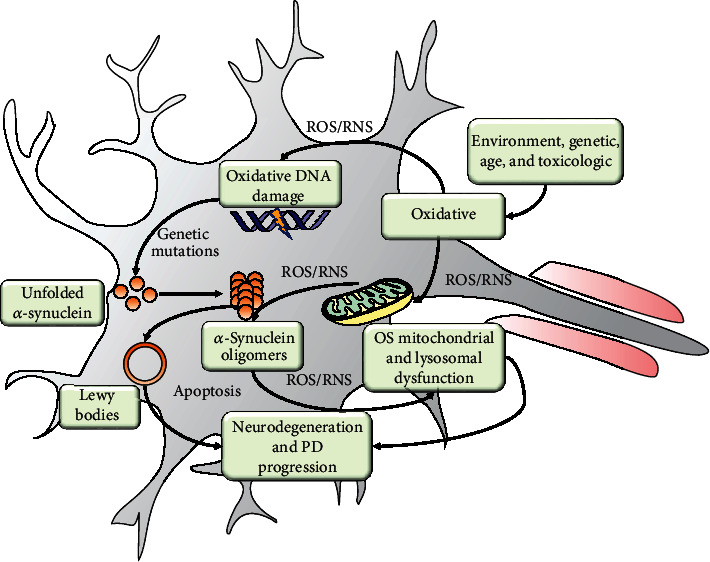
Schematic representation of the oxidative stress mechanism in the development of Parkinson's disease in dopaminergic neurons. Oxidative stress from aging or exogenous sources causes damage to vulnerable cellular structures such as mitochondria and DNA. *α*-Synuclein gene mutations can promote the formation of *α*-synuclein oligomers and Lewis bodies. Oxidative stress causes mitochondrial dysfunction that converts the mitochondria into a source of ROS/RNS. ROS/RNS increases *α*-synuclein aggregate formation, and these, in turn, damage mitochondrial function. Both mitochondrial dysfunction and Lewis bodies lead to a loss of dopaminergic neurons and thus neurodegeneration

ROS are necessary components for cellular homeostasis. However, when ROS are produced in excess, they induce transcription errors that cause dysfunction in the expression of different proteins, including C-terminal *α*-synuclein, parkin, and ubiquitin hydrolase which are directly related to PD [12]. A recent study reported the propensity of oligomers to cause ROS production and significant reduction in the presence of metal chelators such as deferoxamine. This evidence indicates that *α*-synuclein oligomers produce superoxide (O_2_^‐^) radicals that bind to transition metal ions such as copper and iron [13]. *α*-Synuclein toxicity may contribute to elevated cellular oxidative stress. Oxidative stress may trigger *α*-synuclein toxicity [9]. In PD, *α*-synuclein oligomers cause the impairment of proteasomes and lysosomes' degradation activity, increasing protein accumulation and aggregation. The accumulation of *α*-synuclein is associated with a decrease of dopamine release [14]. The mitochondrial respiratory chain can produce oxidative stress by generating ROS and reactive nitrogen species (RNS). Excessive production of ROS and RNS can damage the cell, especially the mitochondrial system. Oxidative stress can trigger apoptosis signaling in nerve cells (Figure 1) [15].

In this review article, we will briefly discuss the role of lipoperoxidation, oxidative damage, DNA repair, mitochondria, endogenous antioxidants, and the anti- and prooxidant effects of some natural foods for daily consumption and some food alternatives with antioxidant potential in PD. These dietary alternatives at low or increased levels can have beneficial or detrimental effects to increase or decrease the signs and symptoms of PD.

### 1.1. Lipoperoxidation in Parkinson's Disease

Oxidative stress induces toxicity in the cell by the oxidation of lipids. Lipid oxidation leads to the accumulation of intracellular aggregates, mitochondrial dysfunction, excitotoxicity, and apoptosis. Oxidative damage is a common phenomenon in neurodegenerative diseases. However, it is unclear whether oxidative stress is a cause or a consequence. The formation of modified lipids via oxidation can produce postmitotic cellular dysfunction, and the dysfunction is capable of leading to necrosis or apoptosis of neurons. Lipoperoxidation of polyunsaturated fatty acids (PUFAs) in cell membranes initiates the cumulative deterioration of cell membrane functions by causing decreased fluidity, reduced electrochemical potential, and increased permeability of the cell membrane [16]. Postmortem studies have shown that the effect of chronic oxidative stress is lipoperoxidation of PUFAs in the SN cell membranes [11]. Malondialdehyde (MDA) and glycosylation end-product levels increase in PD, resulting in impaired oxidation of glucose. The increase of MDA and glycation end-products leads to irreversible oxidation of proteins in the SN and the cerebral cortex. The SN has a high risk of aggressive oxidative attacks via lipoperoxides. It has been previously reported that the distribution of transition metals in the brain showed remarkable regional differences [17]. 4-Hydroxy-2-nonenal is a lipid peroxidation product capable of preventing the fibrillar formation of *α*-synuclein by promoting the formation of secondary *β*-sheets and toxic soluble oligomers in a dose-dependent manner. Therefore, oxidative stress can also influence *α*-synuclein toxicity and mediate the pathogenesis of PD [18]. In the postmortem brains of PD patients, increased carbonylated proteins and TBAR markers have been detected [19]. Lipoperoxidation markers were increased in plasma and cerebrospinal fluid (CSF) in PD patients compared to controls without the disease [20].

### 1.2. Oxidative DNA Damage in Parkinson's Disease

PD is characterized by defects in the ability to repair acute or chronic oxidative damage to neurons [21]. The 8-hydroxy-2′-deoxyguanosine (8-OHdG) marker is an indicator of nucleic acid oxidation; in particular, it is a marker of oxidative damage to nuclear and mitochondrial DNA. In patients with PD, the marker has been found to increase in the CSF coupled with increased levels of oxidized coenzyme Q10 [22].Oxidative DNA damage leads to genomic instability and cellular dysfunction. More than 100 oxidative modifications to DNA are identified; many of these are mutagenic, while others interrupt replication or transcription, leading to cancer or cell death in PD [23]. Oxidative damage can arise from external sources, such as chemical agents and ionizing radiation. However, most of the oxidative damage is caused by ROS produced through normal cellular respiration and metabolism [24]. Oxidative damage to DNA in the brain is particularly frequent since it is produced by endogenous metabolic activity. The continuous electrochemical transmission between brain cells requires a large amount of energy. Brain tissue maintains a high basal metabolic rate to meet high energy demands, resulting in brain cells that produce high levels of ROS [25]. The oxidative stress imbalance amplifies the level of damage within brain cells, increasing the demand for DNA repair activity, requiring additional energy, and creating a perpetual state of oxidative stress. Differentiated postmitotic brain cells lack a robust DNA repair and detection machinery associated with replication [26]. However, brain cells have highly efficient base excision repair (BER) mechanisms to cope with the high oxidative stress involved in neurodegenerative disorders. Emerging research suggests that specific BER pathway deficiencies perpetuate neuronal dysfunction [5, 22]. Injuries that occur in DNA include base modifications, abasic sites, and single- and double-strand breaks of DNA. The injuries that occur are mostly repaired via BER [27]. The first step of BER is the recognition and removal of damaged DNA bases. DNA base modifications are recognized first and removed by glycosylase enzymes. Abasic sites are removed by apurinic enzymes/apyrimidinic endonucleases [28]. DNA glycosylases are the first DNA repair enzymes recruited for oxidative damage [29]. Eleven glycosylases are known in humans [30]. The three central glycosylases that recognize oxidative damage are 8-oxoguanin DNA glycosylase (OGG1), endonuclease III, and endonuclease VIII [29]. OGG1 shows specificity for lesions caused by the oxidative damage marker to DNA 8-oxoguanin. The mutY homolog (MYH) can cleave a mismatched adenine throughout the 8-oxoguanin injury to suppress mutagenicity [31]. In the brain, the most abundant oxidative lesions produced by the 8-oxoguanin and formamidopyrimidine (FAPY G) markers are derived from the oxidation and reduction of 8-hydroxyguanine injuries [32].

### 1.3. Mitochondria in Parkinson's Disease

Mitochondria are organelles that produce ATP (chemical energy) and play a critical role in energy metabolism, the redox state, and Ca^2+^ homeostasis. Therefore, mitochondria are crucial to cell survival. Intracellular Ca^2+^ stimulates the electron transport chain in the mitochondria producing ATP and ROS as subproducts. The endoplasmic reticulum is a quality control organelle that organizes protein synthesis, folding, and transport. Crosstalk between the endoplasmic reticulum and mitochondria increases with oxidative stress and mitochondrial stress, which can cause endoplasmic reticulum dysfunction. Research in the postmortem brain tissue has previously reported impaired mitochondrial function and elevated oxidative stress caused by *α*-synuclein aggregates, autooxidation, and degradation of dopamine in the SN [33]. Chronic oxidative stress is characterized by altered levels of iron and antioxidant defenses (enzyme superoxide dismutase (SOD) and glutathione (GSH)) in brain cells in PD [34]. Antioxidant enzymes, SOD, and GSH prevent ROS levels from rising [35]. When antioxidant defenses fail to regulate ROS levels, there is an increase in OS capable of producing harmful effects [36]. Random oxidation of macromolecules within the cell can damage cell structures and even cause cell death [37]. OS increases the possibility of spontaneous cellular mutations. The appearance of mutations conditions the vulnerability of cells to dysfunction [38].

### 1.4. Prooxidant Foods

**Figure 2:**
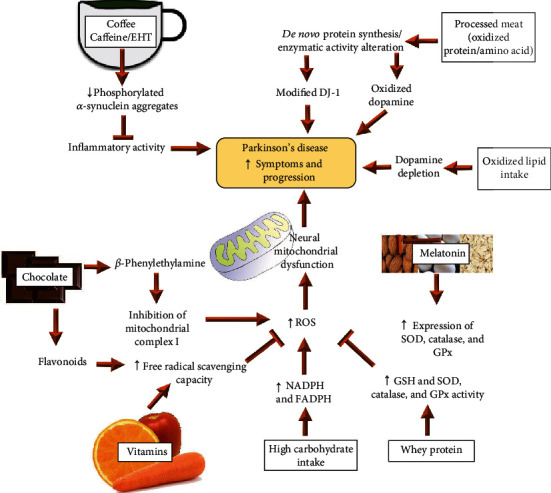
Proposed mechanism of food with pro- and antioxidant properties in the development of PD. Description of how the excessive intake of oxidized proteins and lipids causes the synthesis of oxidized molecules that worsens the symptoms of PD. A diet rich in carbohydrates can increase oxidative stress and cause oxidative neuronal damage. Food products can help to neutralize mediators of PD progression.

Prooxidant foods are compounds that promote oxidative stress by increasing ROS generation or by decreasing antioxidant systems [39]. Diet can participate in OS production processes depending on the quantity or quality of micro- or macronutrients [40]. Some characteristics and mechanisms of different types of prooxidant foods that have the ability to favor the clinical manifestations of PD are described below (Figure 2).

### 1.5. Processed Meat Containing Oxidized Proteins in Parkinson's Disease

Meat products are the primary source of protein, amino acids, vitamins (niacin, vitamin B6, and vitamin B12), and minerals such as iron and zinc [41]. However, meat also contains products that, in excess, can be harmful to human health such as sodium, advanced glycation end-products, cholesterol, and free fatty acids [42, 43]. Currently, most meat products undergo processing stages that involve modification of their structure, changes in aggregation, or fragmentation that can cause protein oxidation [44]. Protein carbonylation determination is a useful marker to measure oxidative damage in different foods with high protein content [45]. Carbonylation is common in some processed foods, such as fermented sausages, dry-cured loins, chicken thigh meat, and pork or beef patties [46]. These products accumulate oxidized molecules during their process, and when ingested, they come into contact with the intestinal mucosa, internal organs, and the bloodstream after intestinal absorption [47]. Various studies have reported that oxidative protein modifications can accumulate in the body and damage specific tissues. For example, protein carbonyls correlated with the severity of damage in inflammatory bowel disease [48], and oxidized thyroxines are associated with dysfunction of insulin secretion [49]. Besides, the proteolytic damage of tissue releases 2-aminoadipic acid, which is a risk marker for diabetes mellitus (DM) [50]. Intake of products with these structural modifications is also associated with aging and age-related diseases like PD [51–53]. There are different pathological mechanisms in which the intake of peptides or modified amino acids in PD is involved. One mechanism depends on the incorporation of oxidized amino acids into *de novo* protein synthesis, resulting in enzyme dysfunction with the ability to cause cellular damage [54]. One of the examples is the oxidative modification of the DJ-1 protein. The DJ-1 protein, which contains 189 amino acids, has been linked to PD because the loss of its functions causes disease with parkinsonian characteristics [55]. The oxidative modifications in a single amino acid of the DJ-1 protein are sufficient to favor PD development. Oxidative modifications of dopamine have been linked to PD [56]. Oxidized dopamine accumulates in the dopaminergic neurons of patients with sporadic or genetic PD, resulting in mitochondrial and lysosomal dysfunction [57].

### 1.6. Oxidized Lipids in Parkinson's Disease

Lipids are a necessary part of nutrition, providing large amounts of energy and essential fatty acids and promoting food acceptance [58]. Lipids provide important quality characteristics to meat products such as flavor and juiciness [59]. Lipids are highly prone to oxidation and represent the leading nonmicrobial cause of decomposition of meat products [60, 61]. During the oxidation of lipids in food, nutrient quality is lost due to the decrease of some macro- and micronutrients such as PUFAs, tocopherols, and amino acids or proteins that react with oxidized lipids [62]. Oxidized cholesterol products can be found in beef, *mortadella*, and anchovies [63–65]. Lipid oxysterols and hydroperoxides can be found in butter, corn oil, or olive oil [66, 67]. During food processing or cooking, lipids can react with proteins to form advanced lipid oxidation end-products (ALE) [68]. The health effects of ALEs in food are controversial. Some authors describe that oxidized fats can activate the inflammatory response and damage organs such as the intestine, liver, and kidney [69, 70]. Baynes reported that enzyme systems can neutralize oxidized fats during metabolism and that harmful metabolic processes can occur in people with compromised cellular functions [71]. The effect of the high-fat diet on PD is not entirely clear. Mouse studies showed that high-fat diets could increase dopamine depletion in the nervous system and promote the progression of PD [72, 73]. Human studies associate the consumption of animal fat with an increased risk of developing PD [74, 75]. However, results were not replicated in other more extensive recent studies [76–78]. The contradictory findings may be due to different types of fat used in the diet that are not always described. Some studies indicate that high cholesterol or a keto diet may lower the risk of developing PD or improve the motor and nonmotor symptoms of the disease [79]. Also, the consumption of PUFAs contributes to the neuroprotective anti-inflammatory capacity [80–82].

### 1.7. The Effect of a Diet Rich in Carbohydrates in Parkinson's Disease

Eating a diet rich in carbohydrates can promote cellular signaling of inflammatory effects [83]. High carbohydrate consumption increases the glycemic index. A high glycemic index is associated with cancer risk and comorbidities due to overweight or obesity [84, 85]. In the Asian population, high consumption of rice or total carbohydrates is positively related to type 2 DM [86, 87]. Additionally, consuming refined carbohydrates, such as fructose-rich syrups, may lead to metabolic problems such as DM, obesity, and cardiovascular disease [88, 89]. High caloric intake can induce oxidative stress by increasing the substrates of mitochondrial respiration [90]. The high level of glucose metabolism increases NADPH and FADPH, which are capable of increasingO_2_^‐^production [91]. High glucose concentrations increase the activity of the thioredoxin-interacting protein that favors the generation of ROS [92]. The increased consumption of fructose products plays an important role as a trigger for oxidative stress. Animal studies showed that increased fructose in the diet causes metabolic and endocrine changes that affect different organs and tissues [93, 94]. Some retrospective studies report risk factors for developing PD due to high carbohydrate intake, but the effects of high carbohydrate intake on PD are still inconclusive. Consumption of dairy products is a risk factor in men, but not in women [74]. It has been previously reported that carbohydrates, monosaccharides, refined sugar, lactose, and other carbohydrate-rich foods such as bread and cereals are risk factors for PD [95]. However, cohort studies have not confirmed that total carbohydrate intake is associated with the risk of developing PD [76]. Diet with a high glycemic index or high carbohydrate intake found in a case-control study reduces the risk of PD [96]. A diet rich in carbohydrates could link DM to the risk of developing PD [97, 98]. PD and DM share common pathogenic mechanisms involving mitochondrial dysfunction, inflammation, and metabolic disturbances [99]. There is evidence between the association of DM with PD risk by increasing postural instability and difficulty walking [100]. However, further confirmatory studies are still needed [101, 102].

### 1.8. Parkinson's Disease Management Alternatives

Due to the increase in life expectancy and generational change, PD has become a common health problem and care of a PD patient has become a treatment challenge. PD is a costly disease for health services characterized by the accelerated appearance of clinical manifestations. PD becomes devastating for patients and their families. In the field of neurodegenerative diseases, the existing therapies only limit the activity of the disease. Alternative treatments need to be evaluated for both their beneficial and harmful properties (Table 1). A combination of therapies is recommended, which could condition the real delay in the evolution of PD as a possibility to improve the symptomatology of the disease or improve the quality of life of patients [103].

### 1.9. Antioxidants in Parkinson's Disease

Cells have developed antioxidant defense systems to protect themselves against their destructive products. The antioxidant defense system consists of enzymes that involve BER, SOD, glutathione peroxidase (GPx), peroxiredoxins, and GSH [26]. Due to the brain's high metabolic rate, there may be a decreased ratio of antioxidant to prooxidant enzymes [104]. The SN's antioxidant defenses are relatively low compared to other regions of the central nervous system (CNS). Low levels of GSH are produced during the early stages of PD. Extravesicular dopamine and its breakdown products can act as GSH depleting agents [105]. N-acetylcysteine (NAC) shows antioxidant properties by restoring cellular GSH and participating in important endogenous antioxidant systems. In experimental studies, NAC has been reported to protect against the development of PD [106]. The antioxidant characteristics of GSH have been demonstrated in oxidative stress models, including models that use buthionine-sulfoximine to deplete GSH. GSH depletion increases oxidative stress in all cells and mitochondrial fractions. Most of the antioxidant functions of GSH are exerted as a cofactor of the GPx enzyme family [107]. The GPx family forms a group of selenium-containing enzymes with the ability to reduce toxic peroxides [108]. Under neurotoxicity conditions, the overexpression of the antioxidant enzyme GPx can decrease the number of neurons lost [109]. An immunocytochemical study of GPx1 expression showed that dopaminergic neurons in SN express low levels of the enzyme. In contrast, in other regions not affected by PD, they express high levels of the enzyme [110]. GPx is an enzyme involved in the elimination of peroxides in the brain. The enzymatic activity of GPx reduces the probability that the hydroxyl radical (OH) will be produced by transition metals [44]. One of the major cellular defense systems for oxidative attacks is the antioxidant enzyme SOD. Three types of SOD have been identified in mammalian cells: copper-zinc SOD (Cu/ZnSOD or SOD1), manganese SOD (MnSOD or SOD2), and extracellular SOD (ECSOD or SOD3). SOD1 is a 32 kDa homodimer of a 153-residue polypeptide with one copper and one zinc-binding site per subunit [111]. Specifically, each monomer possesses a *β*-barrel motif and two functionally important large loops, called zinc and electrostatic loops that coat the metal-binding region. SOD1 catalyzes the reaction of the O_2_^‐^ anion in molecular oxygen (O_2_) and hydrogen peroxide (H_2_O_2_) in a bonded copper ion [112]. The intracellular concentration of SOD1 is high (between 10 and 100 *μ*M) [113]. SOD1 represents 1% of the total protein content in the CNS. SOD1 is located in the cytoplasm, nucleus, lysosomes, peroxisomes, and intermitochondrial membrane spaces of eukaryotic cells [114]. Reports suggest that SOD1 is a crucial antioxidant enzyme whose mutations are a significant target of oxidative damage to brains with PD [115, 116]. SOD1 and mitochondrial SOD2 are among the most abundant antioxidant proteins in the brain and are fundamental in protecting neurons from oxidative stress. SOD enzymes eliminate toxic O_2_^‐^ converting it catalytically into oxygen and H_2_O_2_. Some studies suggest that abnormalities in SOD1 or SOD2 may contribute to the development of PD [117].

### 1.10. Melatonin in Parkinson's Disease

Melatonin is a natural hormone mainly secreted by the pineal gland that regulates different physiological functions. Melatonin is also synthesized by other organisms, such as bacteria, invertebrate animals, and plants [118]. The consumption of foods rich in melatonin such as pineapple, orange, and banana, can increase the antioxidant capacity of the organism [119]. Melatonin has also been identified in vegetables, meats, and sprouts [120, 121]. Meng et al. evaluated that eggs, fish, nuts, cereals, and some seeds are the foods with the highest melatonin content [122]. Melatonin is known for its antioxidant properties and anti-inflammatory and cardiovascular effects. Melatonin has properties that inhibit tumor proliferation in the autoimmune system and provide a neuroprotective effect [123–127]. The main interest in the investigation of the effects of melatonin on PD arises from the relationship between the decrease in the activity of the pineal gland and melatonin in these patients [128]. MT1 and MT2 melatonin receptors are also decreased in PD [129]. Melatonin neutralizes ROS and induces the expression and activity of antioxidant enzymes [130, 131]. In mice, the effect of melatonin counteracts the progression of dopamine by increasing the activity of the mitochondrial complex I by decreasing the levels of lipoperoxides and nitrites in the cytosol and mitochondria of brain cells [132]. Other studies have shown antiapoptotic, neuroprotective, and antidepressant activity in mouse models with PD [133–135]. Other studies have shown that melatonin treatment can help improve sleep disorder and increase neuroprotection in PD patients [136, 137]. However, the consumption of melatonin has not been able to improve the motor symptoms of PD [138]. Some studies reported that melatonin could promote ROS generation. *In vitro* studies showed that melatonin has prooxidant effects mainly at lipids and proteins [139]. However, high concentrations of melatonin (10-1000 *μ*M) are reported to promote ROS production by inducing cytotoxicity and apoptosis in human leukemia cells [140]. Similar effects were found in an Alzheimer's disease model culture, where melatonin concentrations of 1 mM increased oxidative stress markers, while concentrations < 0.1 mM reduced oxidative damage [141]. The prooxidant mechanisms of melatonin have not been fully described. Leukocyte studies show that melatonin has little interaction affinity for calmodulin and that this phenomenon seems to favor ROS production [142]. The benefits and risks of melatonin supplementation in PD patients require more clinical evidence to support the previously described findings.

### 1.11. Vitamins in Parkinson's Disease

Complementary to the usual pharmacological therapy for PD, it is suggested to add some other natural compounds as adjuvants. Vitamins are natural bioactive products with antioxidant properties [135]. Vitamins are necessary to maintain normal body functions; since essential vitamins cannot be synthesized endogenously by the body, they must be obtained through the diet. Vitamin deficiency is common in the elderly. Vitamins A, D, E, and K are fat-soluble. Fat-soluble vitamins bind primarily to nuclear receptors and affect the expression of specific genes [143]. Vitamins B and C are soluble in water and are cofactor constituents that affect enzyme activity [144]. The antioxidant properties of vitamins and their biological functions to regulate gene expression may be beneficial for the treatment of PD. Recent clinical evidence indicates that adequate supplementation of different vitamins can reduce PD incidence and improve the clinical symptoms of patients. Vitamin supplementation may be a beneficial adjuvant treatment for PD [145]. The members of the vitamin B family which are soluble in water include thiamine (vitamin B1), riboflavin (vitamin B2), niacin (vitamin B3), pantothenic acid (vitamin B5), pyridoxine (vitamin B6), biotin (vitamin B7), folic acid (vitamin B9), and cobalamin (vitamin B12) [146]. B vitamins play an important role as enzyme cofactors in multiple biochemical pathways in all tissues, such as regulating metabolism, improving the immune system and nervous system function, and promoting growth and cell division. Recently, the association between vitamin B and PD is receiving increasing attention [147]. Fukushima suggests that excess vitamin B3 (nicotinamide) is related to PD development [148]. Excess nicotinamide can induce the overproduction of 1-methyl nicotinamide (MNA) in PD patients [149]. Griffin et al. found that the singular form of nicotinamide (10 mM) has a significant effect in inducing the differentiation of embryonic stem cells in neurons. However, high singular forms (>20 mM) of nicotinamide cause cytotoxicity and cell death [150].

Vitamin C (ascorbic acid) is an essential water-soluble vitamin that is widely distributed in various tissues. Vitamin C is abundant in vegetables, fresh fruits, and animal livers. Vitamin C contains two molecular subforms in the body. The reduced form of vitamin C is ascorbic acid and the oxidized form of dehydroascorbic acid. Vitamin C is essential for the nervous system's physiological function and antioxidant function by inhibiting oxidative stress, reducing lipoperoxidation, and eliminating free radicals [151]. Vitamin C has the potential for the treatment of PD because it is mainly distributed in areas rich in neurons [152]. Vitamin C deficiency can cause scurvy. However, ascorbic acid exhibits prooxidant properties in the presence of free transition metals because it reduces ferric ions to ferrous ions in a Fenton-type reaction. Ascorbic acid in the presence of H_2_O_2_ stimulates the formation of OH radicals [153]. Therefore, the final prooxidant or antioxidant effect depends on the relationship between ascorbic acid concentration and the available ferrions [38]. At sufficiently high levels, ascorbic acid reduces and destroys the radicals formed [154]. Postmortem studies have shown that vitamin D receptors are present in dopaminergic neurons in the human SN. Vitamin D administration has been suggested to protect dopaminergic neurons, and its deficiency is associated with increased motor severity, postural instability, worsening verbal fluency, and memory [155, 156]. In early PD, patients have been reported to have significantly lower serum 25-(OH)-D concentrations than controls of the same age, which may have implications for bone health and fracture risk. Sleeman et al. reported a small significant association between vitamin D status at baseline and worsening PD motor function at 36-month follow-up [157]. 25-Hydroxyvitamin-D deficiency and reduced exposure to sunlight are significantly associated with an increased risk of PD. However, vitamin D supplementation does not produce significant benefits to improve motor function in PD patients [158].

### 1.12. Whey Protein Supplementation in Parkinson's Disease

In addition to the protein obtained from food, there is also whey protein (WP) supplements used to treat some metabolic disorders [159]. WP is a soluble by-product obtained from the separation of casein during cheesemaking [160]. WP is mainly rich in globulins, albumin, and amino acids [161, 162]. Some studies have shown that specific WP preparations can reduce proinflammatory cytokine levels (TNF-*α*, IL-6) and work as a hepatoprotective agent in hepatitis and liver fibrosis in rat models [163]. Other studies evaluated the antioxidant effect of (WP) supplementation on oxidative stress [164]. Rat studies reported that WP supplementation increases the antioxidant enzyme activity of catalase, SOD, and GPx and reduces the effect of TBAR [165]. Falim et al. evaluated the effect of WP supplementation on oxidative stress in subjects with overweight/obesity and DM. The authors found no significant effect on oxidative stress markers (TBAR, AOPP, and 8-OHdG) [166]. Reyes et al. and Katz et al. demonstrated that supplementation of the amino acid NAC contributes to raising GSH levels in mice and patients with PD [167, 168]. PD patients are generally malnourished and have decreased muscle strength. In these patients, the use of WP may be recommended [169–171], although there is little clinical evidence in this regard. Tosukhowong et al. conducted a double-blind, placebo-controlled clinical trial of 38 patients with PD, and they also conducted a six-month follow-up to assess WP's clinical effects. The authors found that 20 g/day increased the levels of reduced GSH and decreased homocysteine levels. These results did not impact the severity of PD measured according to the unified PD classification scale (UPDRS) [172]. Clinical studies involving a larger number of patients with long-term follow-up are required to establish the possible beneficial effects of WP supplementation in PD patients. WP supplementation must be monitored because high protein intake decreases the therapeutic effects of levodopa, increasing the symptoms of PD [173].

### 1.13. Chocolate in Parkinson's Disease

Chocolate is produced from the cocoa bean of the *Theobroma cacao* tree. Polyphenols, especially flavonoids, are the main components of health interest in cocoa and its derivatives [174]. Currently, research on the potential health benefits of consuming PD cocoa is attractive due to their high content of antioxidant polyphenols [175]. The antioxidant capacity of flavonoids has been previously reported due to their free radical scavenging capacity, chelation of transition metal ions, and the mediation of some cell signaling cascades [176]. *In vitro* studies have shown the beneficial antioxidant effects of cocoa [177, 178]. However, these effects are not always extrapolated to *in vivo* studies [179]. It is difficult to establish the recommended amount of chocolate intake to obtain any specific health benefit because the bioavailability characteristics and polyphenol contents are different in each type of chocolate [180]. Dark chocolate, unlike white or milk chocolate, has been used in studies to evaluate its health effects due to its high flavonoid content close to 50% [181]. Dark chocolate shows potential benefits in DM [182], cancer [183], cardiovascular disease [184], and neuroprotective effects [185]. The study on the effects of chocolate in PD patients could be of great importance because the intake of chocolate and other sweets is frequent. A study reported that PD patients consume more chocolate (100 g weekly) than a control group without the disease. PD patients increase chocolate intake by 22% during the disease [186]. A single-dose crossover study evaluated the immediate effect of 200 g of cocoa chocolate on motor function in PD patients. Contrary to expectations, no significant differences in motor function were found in this study at 1-3 h after the ingestion of cocoa chocolate compared to cocoa-free chocolate [187]. An important factor to consider in addition to the flavonoid content is that cocoa contains *β*-phenylethylamine, traces of a type of amine with neurotransmitter activity [188, 189]. Studies suggest that the distribution of *β*-phenylethylamine in the brain reaches its maximum concentration in dopaminergic regions [190]. Studies in mice show that *β*-phenylethylamine causes inhibition of mitochondrial complex I favoring the generation of OH and psychomotor dysfunction [191]. Furthermore, the intake of *β*-phenylethylamine in mice causes alterations of akinesia, catalepsy, and other motor disorders found in PD [192, 193]. Due to this, it has been reported that long-term intake of cocoa chocolate can promote neurodegeneration and dopamine complications due to its content of *β*-phenylethylamine [194]. There is still insufficient clinical evidence to support the benefits of the chocolate diet in PD patients. It is necessary to know the singular form and composition of cocoa-derived products that can help improve PD symptoms safely.

### 1.14. Coffee in Parkinson's Disease

Normally, soluble *α*-synuclein in PD is intrinsically disordered. This protein erroneously folds and forms distinctive amyloid fibrils in neuropathological inclusions. Initial oligomerization and eventual fibrillation are believed to be critical steps leading to neuronal dysfunction and death [195]. Postmortem brain studies show that *α*-synuclein in aggregates is hyperphosphorylated in serine 129, and antibodies to phospho-Ser129-*α*-synuclein (p-*α*-syn) are useful in detecting these inclusions [196]. The phosphorylation of *α*-synuclein in serine 129 accelerates its oligomerization and fibrillation *in vitro*. Consequently, this posttranslational modification is of pathogenic and therapeutic interest in *α*-synucleinopathies [197]. Dephosphorylation of the protein is carried out by a specific isoform of protein phosphatase 2A (PP2A). Serine/threonine phosphatase is the primary brain enzyme consisting of a structural A subunit, a catalytic C subunit, and one of the multiple regulatory B subunits that determine substrate specificity [198]. Carboxyl PP2A methylation is regulated by different PP2A-specific leucine carboxyl methyltransferase 1 (LCMT-1) and a PP2A-specific methyl-esterase (PME-1). The levels of these methylation regulatory enzymes are disturbed in the brains in PD, with low regulation of LCMT-1 and high regulation of PME-1, associated with reduced relative levels of methylated PP2A (methyl-PP2A), which is the enzymatically more active form [199]. In addition to the antioxidant effects present in the components of coffee [200], caffeine has shown protective effects on altered *α*-synuclein activity in PD [201]. In 2013, the treatment of transgenic mice with PD was reported; these mice were administered eicosanoyl-5-hydroxytryptamide (EHT), an inhibitor of PME-1 methyl esterase activity present in many types of coffee. The authors found increased brain methylation and PP2A phosphatase activity with reduced accumulation of phosphorylated *α*-synuclein aggregates with improved neuronal integrity and suppression of the neuroinflammatory response [202]. The study results suggest that EHT and caffeine have synergistic effects in protecting the brain against *α*-synuclein-mediated toxicity by maintaining active PP2A [203]. Decaffeinated coffee has even been found to have a protective effect in PD models in *Drosophila* [204]. Caffeine is one of the widely consumed purines (phytochemicals) that can contribute beneficial effects to the brain. Among the purines, caffeine is the most studied, theobromine and theophylline have been studied less, and other methylxanthines have been mostly unexplored. While caffeine's neurological effects are well established, it is unknown whether this purine alone is responsible for the beneficial effects of coffee consumption on cognition and resistance to neurodegenerative disorders. Emerging evidence suggests that other classes of phytochemicals present in large quantities in coffee may improve neuroplasticity and protect neurons against dysfunction and degeneration. Among the many nonpurine phytochemicals in coffee, flavonoids such as epicatechins have been shown to promote synaptic plasticity [205]. Growing evidence indicates that regular coffee consumption results in better cognitive performance during stressful conditions [206]. Acute caffeine intake improves performance on memory tasks [207]. A 150 mg dose of caffeine was previously reported to improve cognitive performance for at least 10 h, and caffeine is recommended in military rations [208]. Extensive longitudinal clinical studies have established an inverse relationship between coffee consumption and decreased memory during aging [209].

### 1.15. Other Phytochemicals in Parkinson's Disease

Interest in scientific research on the properties of natural antioxidants in chronic degenerative diseases has been steadily increasing over the past two decades [210]. Medicinal plants are the source of a wide variety of bioactive components with antioxidant and anti-inflammatory properties that can be useful as neuroprotective agents [211]. The neuroprotective molecular mechanisms of plant extracts include the elimination of toxins, antioxidant activity, and antiapoptotic effects [212, 213]. Berberine is one of the active components of different Chinese medicinal herbs, including *Hydrastis canadensis*, *Coptis chinensis*, *Berberis aquifolium*, and *Berberis vulgaris* [214]. Berberine has been used as a natural remedy to treat diarrhea, stomatitis [215], hepatitis [216], and hypoglycemic effect [217]. Studies have shown that berberine also has neuroprotective effects by regulating neurotrophin levels [218, 219]. Experimental PD models report that berberine prevents the loss of dopaminergic neurons and enhances motor balance and coordination with maximum effect at 50 mg/kg [220]. However, the long-term treatment with berberine has been associated with decreased dopamine levels and increased degeneration of dopaminergic neuronal cells and its loss in experimental models of PD in rats [221, 222].

Curcumin is a compound derived from the *Curcuma longa* plant that has been extensively researched for its antioxidant and anti-inflammatory properties [223]. *In vitro* studies report that the antioxidant properties of curcumin contribute to its neuroprotective effects [224]. Cognitive deficiencies have improved after the administration of curcumin due to increased levels of the brain-derived neurotrophic factor [225]. Curcumin also has beneficial effects on PD by destabilizing the *α*-synuclein protein [226]. A study in the *Drosophila* model of PD has shown that the administration of curcumin decreases ROS and neurodegenerative severity and improves motor skills [227].

Another bioactive molecule of natural products is quercetin. Quercetin is one of the main flavonoids widely distributed in apples, berries, onions, tea, tomatoes, and other plant products [228]. The antioxidant and anti-inflammatory properties of quercetin administration have been demonstrated in rat models [229, 230]. The administration of quercetin and piperine (a natural alkaloid) has potent neuroprotective effects against neurotoxicity in rat PD models [231].

## 2. Conclusions

PD is a common neurodegenerative disorder. PD incidence generally increases with age. Potential risk factors in developing PD include environmental toxins, drugs, pesticides, brain microtrauma, focal cerebrovascular damage, and genomic defects. Previous studies suggest that the intake of certain products may be associated with an increased risk of PD. Many foods for daily consumption have benefits because of their content of amino acids, vitamins, minerals, and micronutrients. However, increased use of some prooxidant foods may increase the risk of developing or increasing PD symptoms. Processed meat is characterized by high sodium content, advanced glycation end-products, cholesterol, and free fatty acids. The overconsumption of meat conditions a prooxidant effect. The alteration or fragmentation of the structure of meat products can cause oxidation of proteins by carbonylation. On the other hand, fruits and vegetables stand out for their antioxidant effect due to their amounts of vitamins and minerals. The evidence about the beneficial effect of coffee intake and the health risk by consuming large amounts of chocolate in PD patients is noteworthy. Short-term, medium-term, and long-term follow-up clinical studies are required to establish the useful quantities of the food substances, alone or in combination, to determine the bioavailability and nutritional content of each type of food in PD.

## Figures and Tables

**Table 1:** Antioxidant and prooxidant properties of nutrients used in Parkinson's disease.

Nutrient	Antioxidant/benefit effects in PD	Prooxidant/side effects in PD
*Melatonin*	Increases the expression of GPx, SOD, and catalase [134] Improves sleep disturbances in PD patients [139, 140]	Melatonin can promote ROS production at a concentration of 10-1000 *μ*M [143]

*Vitamins*	The low singular form of vitamin B (10 mM) can induce differentiation of embryonic stem neuron cells [153] Vitamin C has antioxidant properties and it is well distributed in the brain [155] Vitamin D protects dopaminergic neurons [159]	The high singular form of vitamin B3 (>20 mM) can induce cytotoxicity and cell death [153] Vitamin C can induce OS in the presence of free transition metals and H_2_O_2_ [156]

*Whey protein supplements*	20 g/day increases GSH in PD patients but does not improve the severity of disease [176]	High protein intake decreases the absorption of levodopa and increases the symptoms of PD [177]

*Chocolate*	Chocolate rich in flavonoids has free radical scavenging capacity and neuroprotective effects [180] No improvement was found in motor function after administration of 200 g of cocoa chocolate in PD patients [188]	Cocoa chocolate contains *β*-phenylethylamine which can promote ^−^OH formation and psychomotor dysfunction [192]

*Berberine*	Administration of 50 mg/kg prevents loss of dopaminergic neurons and improves motor balance and coordination in a rat PD model [219]	Long-term administration of berberine increases loss of dopaminergic neuronal mass *in vitro* and *in vivo* [220] Berberine along with chronic L-DOPA administration causes degeneration of dopaminergic cells in the substantia nigra in a rat model of PD [221]

*Curcumin*	Decreases ROS and the neurodegenerative severity and improves locomotor symptoms in *Drosophila* PD model [226]	

*Quercetin*	Administration of quercetin and piperine decreases the neurotoxicity in rat PD model [230]	

*Coffee*	Components in coffee have antioxidant, anti-inflammatory, and neuroprotective effects [204, 206]	

GSH: glutathione; GPx: glutathione peroxidase; SOD: superoxide dismutase; PD: Parkinson's disease; ROS: reactive oxygen species.

## References

[1] Mayo J. C., Sainz R. M., Tan D. X., Antolín I., Rodríguez C., Reiter R. J. (2005). Melatonin and Parkinson’s disease. *Endocrine*.

[2] Emamzadeh F. N., Surguchov A. (2018). Parkinson’s disease: biomarkers, treatment, and risk factors. *Frontiers in Neuroscience*.

[3] Ahmed H., Abushouk A. I., Gabr M., Negida A., Abdel-Daim M. M. (2017). Parkinson's disease and pesticides: A meta-analysis of disease connection and genetic alterations. *Biomedicine & Pharmacotherapy*.

[4] García S., López B., DEG M., OAJ V., Coral V. R. (2010). Breve reseña histórica de la enfermedad de Parkinson. De la descripción precipitada de la enfermedad en el siglo XIX, a los avances en biología molecular del padecimiento. *Medicina Interna de México*.

[5] Bellucci A., Mercuri N. B., Venneri A. (2016). Review: Parkinson's disease: from synaptic loss to connectome dysfunction. *Neuropathology and Applied Neurobiology*.

[6] Bellucci A., Zaltieri M., Navarria L., Grigoletto J., Missale C., Spano P. (2012). From *α*-synuclein to synaptic dysfunctions: New insights into the pathophysiology of Parkinson's disease. *Brain Research*.

[7] Bartels T., Choi J. G., Selkoe D. J. (2011). *α*-Synuclein occurs physiologically as a helically folded tetramer that resists aggregation. *Nature*.

[8] Volles M. J., Lansbury P. T. (2003). Zeroing in on the pathogenic form of alpha-synuclein and its mechanism of neurotoxicity in Parkinson’s disease. *Biochemistry*.

[9] Barzilai A., Melamed E. (2003). Molecular mechanisms of selective dopaminergic neuronal death in Parkinson's disease. *Trends in Molecular Medicine*.

[10] Tabbal S. D., Tian L., Karimi M., Brown C. A., Loftin S. K., Perlmutter J. S. (2012). Low nigrostriatal reserve for motor parkinsonism in nonhuman primates. *Experimental Neurology*.

[11] Puspita L., Chung S. Y., Shim J. W. (2017). Oxidative stress and cellular pathologies in Parkinson’s disease. *Molecular Brain*.

[12] Ortiz G. G., Pacheco-Moisés F. P., Gómez-Rodríguez V. M., González-Renovato E. D., Torres-Sánchez E. D., Ramírez-Anguiano A. C. (2013). Fish oil, melatonin and vitamin E attenuates midbrain cyclooxygenase-2 activity and oxidative stress after the administration of 1-methyl-4-phenyl-1,2,3,6- tetrahydropyridine. *Metabolic Brain Disease*.

[13] Dexter D. T., Wells F. R., Agid F. (1987). Increased Nigral Iron Content in Postmortem Parkinsonian Brain. *The Lancet*.

[14] Melo T. Q., Copray S. J. C. V. M., Ferrari M. F. R. (2018). Alpha-synuclein toxicity on protein quality control, mitochondria and endoplasmic reticulum. *Neurochemical Research*.

[15] Ortiz G. G., Crespo-López M. E., Morán-Moguel C., García J. J., Reiter R. J., Acuña-Castroviejo D. (2001). Protective role of melatonin against MPTP-induced mouse brain cell DNA fragmentation and apoptosis in vivo. *Neuro endocrinology letters*.

[16] Dexter D. T., Carter C. J., Wells F. R. (1989). Basal lipid peroxidation in substantia nigra is increased in Parkinson’s disease. *Journal of Neurochemistry*.

[17] Ortiz G. G., Pacheco-Moisés F. P., Mireles-Ramírez M. A., Ahmad R. (2016). Oxidative stress and Parkinson’s disease: effects on environmental toxicology. *Free Radicals and Diseases*.

[18] Qin Z., Hu D., Han S., Reaney S. H., Di Monte D. A., Fink A. L. (2007). Effect of 4-hydroxy-2-nonenal modification on alpha-synuclein aggregation. *The Journal of Biological Chemistry*.

[19] Navarro A., Boveris A., Bández M. J. (2009). Human brain cortex: mitochondrial oxidative damage and adaptive response in Parkinson disease and in dementia with Lewy bodies. *Free Radical Biology and Medicine*.

[20] Buhmann C., Arlt S., Kontush A. (2004). Plasma and CSF markers of oxidative stress are increased in Parkinson's disease and influenced by antiparkinsonian medication. *Neurobiology of Disease*.

[21] Canugovi C. H., Misiak M., Ferrarelli L. K., Croteau D. L., Bohr V. A. (2013). The role of DNA repair in brain related disease pathology. *DNA Repair*.

[22] Isobe C., Abe T., Terayama Y. (2010). Levels of reduced and oxidized coenzymeQ-10 and 8-hydroxy-2’-deoxyguanosine in the cerebrospinal fluid of patients with living Parkinson’s disease demonstrate that mitochondrial oxidative damage and/or oxidative DNA damage contributes to the neurodegenerative process. *Neuroscience Letters*.

[23] Dizdaroglu M. (2005). Base-excision repair of oxidative DNA damage by DNA glycosylases. *Mutation Research/Fundamental and Molecular Mechanisms of Mutagenesis*.

[24] Hoeijmakers J. H. J. (2001). Genome maintenance mechanisms for preventing cancer. *Nature*.

[25] Floyd R., Hensley K. (2002). Oxidative stress in brain aging: Implications for therapeutics of neurodegenerative diseases. *Neurobiology of Aging*.

[26] Barnes D. E., Lindahl T. (2004). Repair and genetic consequences of endogenous DNA base damage in mammalian cells. *Annual Review of Genetics*.

[27] Wilson D. M., Bohr V. A. (2007). The mechanics of base excision repair, and its relationship to aging and disease. *DNA Repair*.

[28] Dianov G. L., Sleeth K. M., Dianova I. I., Allinson S. L. (2003). Repair of abasic sites in DNA. *Mutation Research/Fundamental and Molecular Mechanisms of Mutagenesis*.

[29] Hegde M. L., Hazra T. K., Mitra S. (2008). Early steps in the DNA base excision/single-strand interruption repair pathway in mammalian cells. *Cell Research*.

[30] Hu J., de Souza-Pinto N. C., Haraguchi K. (2005). Repair of formamidopyrimidines in DNA involves different glycosylases: role of the OGG1, NTH1, and NEIL1 enzymes. *The Journal of Biological Chemistry*.

[31] Ide H., Kotera M. (2004). Human DNA glycosylases involved in the repair of oxidatively damaged DNA. *Biological & Pharmaceutical Bulletin*.

[32] Kalam M. A., Haraguchi K., Chandani S. (2006). Genetic effects of oxidative DNA damages: comparative mutagenesis of the imidazole ring-opened formamidopyrimidines (Fapy lesions) and 8-oxo-purines in simian kidney cells. *Nucleic Acids Research*.

[33] Dauer W., Przedborski S. (2003). Parkinson's Disease: Mechanisms and Models. *Neuron*.

[34] Benzi G., Moretti A. (1995). Are reactive oxygen species involved in Alzheimer's disease?. *Neurobiology of Aging*.

[35] Indo H. P., Yen H. C., Nakanishi I. (2015). A mitochondrial superoxide theory for oxidative stress diseases and aging. *Journal of Clinical Biochemistry and Nutrition*.

[36] Rego A. C., Oliveira C. R. (2003). Mitochondrial dysfunction and reactive oxygen species in excitotoxicity and apoptosis: implications for the pathogenesis of neurodegenerative diseases. *Neurochemical Research*.

[37] Wiseman H., Halliwell B. (1996). Damage to DNA by reactive oxygen and nitrogen species: role in inflammatory disease and progression to cancer. *Biochemical Journal*.

[38] Floor E., Wetzel M. G. (1998). Increased protein oxidation in human substantia nigra pars compacta in comparison with basal ganglia and prefrontal cortex measured with an improved dinitrophenylhydrazine assay. *Journal of Neurochemistry*.

[39] Rahal A., Kumar A., Singh V. (2014). Oxidative Stress, Prooxidants, and Antioxidants: The Interplay. *BioMed Research International*.

[40] Tan B. L., Norhaizan M. E., Liew W.-P.-P. (2018). Nutrients and oxidative stress: friend or foe?. *Oxidative Medicine and Cellular Longevity*.

[41] de Castro Cardoso P. M., dos Reis Baltazar Vicente P. A. F. (2013). Meat nutritional composition and nutritive role in the human diet. *Meat Science*.

[42] Willerson J. T., Ridker P. M. (2004). Inflammation as a cardiovascular risk factor. *Circulation*.

[43] Nowotny K., Schröter D., Schreiner M., Grune T. (2018). Dietary advanced glycation end products and their relevance for human health. *Ageing Research Reviews*.

[44] Kaur L., Maudens E., Haisman D. R., Boland M. J., Singh H. (2014). Microstructure and protein digestibility of beef: the effect of cooking conditions as used in stews and curries. *LWT - Food Science and Technology*.

[45] Fedorova M., Bollineni R. C., Hoffmann R. (2014). Protein carbonylation as a major hallmark of oxidative damage: update of analytical strategies. *Mass Spectrometry Reviews*.

[46] Soladoye O. P., Juarez M. L., Aalhus J. L., Shand P., Estevez M. (2015). Protein oxidation in processed meat: mechanisms and potential implications on human health. *Comprehensive Reviews in Food Science and Food Safety*.

[47] Estévez M., Luna C. (2017). Dietary protein oxidation: a silent threat to human health?. *Critical Reviews in Food Science and Nutrition*.

[48] Keshavarzian A., Banan A., Farhadi A. (2003). Increases in free radicals and cytoskeletal protein oxidation and nitration in the colon of patients with inflammatory bowel disease. *Gut*.

[49] Ding Y.-Y., Li Z.-Q., Cheng X.-R. (2017). Dityrosine administration induces dysfunction of insulin secretion accompanied by diminished thyroid hormones T_3_ function in pancreas of mice. *Amino Acids*.

[50] Wang T. J., Ngo D., Psychogios N. (2013). 2-Aminoadipic acid is a biomarker for diabetes risk. *Journal of Clinical Investigations*.

[51] Shacter E. (2000). Quantification and significance of protein oxidation in biological samples. *Drug Metabolism Reviews*.

[52] Butterfield D. A., Lauderback C. M. (2002). Lipid peroxidation and protein oxidation in Alzheimer's disease brain: potential causes and consequences involving amyloid *β*-peptide-associated free radical oxidative stress 1, 2. *Free Radical Biology and Medicine*.

[53] Stadtman E. R. (2009). Protein oxidation and aging. *Free radical research*.

[54] Gurer-Orhan H., Ercal N., Mare S., Pennathur S., Orhan H., Heinecke J. W. (2006). Misincorporation of free *m*-tyrosine into cellular proteins: a potential cytotoxic mechanism for oxidized amino acids. *Biochemical Journal*.

[55] Repici M., Giorgini F. (2019). DJ-1 in Parkinson’s disease: clinical insights and therapeutic perspectives. *Journal of Clinical Medicine*.

[56] Choi J., Sullards M. C., Olzmann J. A. (2006). Oxidative damage of DJ-1 is linked to sporadic Parkinson and Alzheimer diseases. *Journal of Biological Chemistry*.

[57] Burbulla L. F., Song P., Mazzulli J. R. (2017). Dopamine oxidation mediates mitochondrial and lysosomal dysfunction in Parkinson’s disease. *Science*.

[58] Meynier A., Genot C. (2017). Molecular and structural organization of lipids in foods: their fate during digestion and impact in nutrition. *OCL, Oilseeds Fats Crops and Lipids*.

[59] Wood J. D., Richardson R. I., Nute G. R. (2004). Effects of fatty acids on meat quality: a review. *Meat Science*.

[60] Min B., Ahn D. U. (2005). Mechanism of lipid peroxidation in meat and meat products—a review. *Food Science Biotechnology*.

[61] Lorenzo J. M., Gómez M. (2012). Shelf life of fresh foal meat under MAP, overwrap and vacuum packaging conditions. *Meat Science*.

[62] Eder K., Ringseis R., Decker E. A., Elias R. J., McClements D. J. (2010). Health aspects of oxidized dietary fats. *Oxidation in Foods and Beverages and Antioxidant Applications. Understanding Mechanisms of Oxidation and Antioxidant Activity*.

[63] Boselli E., Rodriguez-Estrada M. T., Fedrizzi G., Caboni M. F. (2009). Cholesterol photosensitised oxidation of beef meat under standard and modified atmosphere at retail conditions. *Meat Science*.

[64] Novelli E., Zanardi E., Ghiretti G. P. (1998). Lipid and cholesterol oxidation in frozen stored pork, salame milano and mortadella. *Meat Science*.

[65] Shozen K., Ohshima T., Ushio H., Takiguchi A., Koizumi C. (1997). Effects of antioxidants and packing on cholesterol oxidation in processed anchovy during storage. *LWT - Food Science and Technology*.

[66] Pie J. E., Spahis K., Seillan C. (1990). Evaluation of oxidative degradation of cholesterol in food and food ingredients: identification and quantification of cholesterol oxides. *Journal of Agricultural and Food Chemistry*.

[67] Udilova N., Jurek D., Marian B., Gille L., Schulte-Hermann R., Nohl H. (2003). Induction of lipid peroxidation in biomembranes by dietary oil components. *Food and Chemical Toxicology*.

[68] Pamplona R. (2011). Advanced lipoxidation end-products. *Chemico-Biological Interactions*.

[69] Koschinsky T., He C. J., Mitsuhashi T. (1997). Orally absorbed reactive glycation products (glycotoxins): an environmental risk factor in diabetic nephropathy. *Proceedings of the National Academy of Sciences*.

[70] Kanner J. (2007). Dietary advanced lipid oxidation end-products are risk factors to human health. *Molecular Nutrition & Food Research*.

[71] Baynes J. W. (2007). Dietary ALEs are a risk to human health-NOT!. *Molecular Nutrition & Food Research*.

[72] Morris J. K., Bomhoff G. L., Stanford J. A., Geiger P. C. (2010). Neurodegeneration in an animal model of Parkinson’s disease is exacerbated by a high-fat diet. *American Journal of Physiology-Regulatory, Integrative and Comparative Physiology*.

[73] Bousquet M., St-Amour I., Vandal M., Julien P., Cicchetti F., Calon F. (2012). High-fat diet exacerbates MPTP-induced dopaminergic degeneration in mice. *Neurobiology of Disease*.

[74] Anderson C., Checkoway H., Franklin G. M., Beresford S., Smith-Weller T., Swanson P. D. (1999). Dietary factors in Parkinson’s disease: the role of food groups and specific foods. *Movement Disorders*.

[75] Chen H., Zhang S. M., Hernan M. A., Willett W. C., Ascherio A. (2002). Diet and Parkinson’s disease: a potential role of dairy products in men. *Annals of Neurology*.

[76] Powers K. M., Smith-Weller T., Franklin G. M., Longstreth W. T., Swanson P. D., Checkoway H. (2003). Parkinson’s disease risks associated with dietary iron, manganese, and other nutrient intakes. *Neurology*.

[77] Chen H., Zhang S. M., Hernán M. A., Willett W. C., Ascherio A. (2003). Dietary intakes of fat and risk of Parkinson’s disease. *American Journal of Epidemiology*.

[78] Dong J., Beard J. D., Umbach D. M. (2014). Dietary fat intake and risk for Parkinson’s disease. *Movement Disorders*.

[79] Phillips M. C. L., Murtagh D. K. J., Gilbertson L. J., Asztely F. J. S., Lynch C. D. P. (2018). Low-fat versus ketogenic diet in Parkinson’s disease: a pilot randomized controlled trial. *Movement Disorders*.

[80] Hernando S., Requejo C., Herran E. (2019). Beneficial effects of n-3 polyunsaturated fatty acids administration in a partial lesion model of Parkinson's disease: The role of glia and NRf2 regulation. *Neurobiology of Disease*.

[81] Kim H. Y., Akbar M., Kim K. Y. (2001). Inhibition of neuronal apoptosis by polyunsaturated fatty acids. *Journal of Molecular Neuroscience*.

[82] Mori M. A., Delattre A. M., Carabelli B. (2017). Neuroprotective effect of omega-3 polyunsaturated fatty acids in the 6-OHDA model of Parkinson’s disease is mediated by a reduction of inducible nitric oxide synthase. *Nutritional Neuroscience*.

[83] Piya M. K., McTernan P. G., Kumary S. (2013). Adipokine inflammation and insulin resistance: the role of glucose, lipids and endotoxin. *Journal of Endocrinology*.

[84] Turati F., Galeone C., Gandini S. (2015). High glycemic index and glycemic load are associated with moderately increased cancer risk. *Molecular Nutrition & Food Research*.

[85] Schwingshackl L., Hoffmann G. (2013). Long-term effects of low glycemic index/load vs. high glycemic index/load diets on parameters of obesity and obesity-associated risks: a systematic review and meta-analysis. *Nutrition, Metabolism and Cardiovascular Diseases*.

[86] Hu E. A., Pan A., Malik V., Sun Q. (2012). White rice consumption and risk of type 2 diabetes: meta-analysis and systematic review. *BMJ*.

[87] Nanri A., Mizoue T., Noda M. (2010). Rice intake and type 2 diabetes in Japanese men and women: the Japan Public Health Center-based prospective study. *American Journal of Clinical Nutrition*.

[88] DiNicolantonio J. J., O'Keefe J. H., Lucan S. C. (2015). Added Fructose: A Principal Driver of Type 2 Diabetes Mellitus and Its Consequences. *Mayo Clinic Proceedings*.

[89] Hu F. B., Malik V. S. (2010). Sugar-sweetened beverages and risk of obesity and type 2 diabetes: epidemiologic evidence. *Physiology & Behavior*.

[90] Teodoro J. S., Duarte F. V., Gomes A. P. (2013). Berberine reverts hepatic mitochondrial dysfunction in high-fat fed rats: a possible role for SirT3 activation. *Mitochondrion*.

[91] Brownlee M. (2005). The pathobiology of diabetic complications: a unifying mechanism. *Diabetes*.

[92] Shah A., Xia L., Goldberg H., Lee K. W., Quaggin S. E., Fantus I. G. (2013). Thioredoxin-interacting protein mediates high glucose-induced reactive oxygen species generation by mitochondria and the NADPH oxidase, Nox4, in mesangial cells. *Journal of Biological Chemistry*.

[93] Alzamendi A., Giovambattista A., Raschia A. (2009). Fructose-rich diet-induced abdominal adipose tissue endocrine dysfunction in normal male rats. *Endocrine*.

[94] Francini F., Castro M. C., Schinella G. (2010). Changes induced by a fructose-rich diet on hepatic metabolism and the antioxidant system. *Life Sciences*.

[95] Ishihara L., Brayne C. (2005). A systematic review of nutritional risk factors of Parkinson’s disease. *Nutrition Research Reviews*.

[96] Murakami K., Miyake Y., Sasaki S. (2010). Dietary glycemic index is inversely associated with the risk of Parkinson's disease: A case-control study in Japan. *Nutrition*.

[97] AlEssa H. B., Bhupathiraju S. N., Malik V. S. (2015). Carbohydrate quality and quantity and risk of type 2 diabetes in US women. *The American Journal of Clinical Nutrition*.

[98] Oba S., For the Japan Public Health Center-based Prospective Study Group, Nanri A. (2013). Dietary glycemic index, glycemic load and incidence of type 2 diabetes in Japanese men and women: the Japan Public Health Center-based prospective study. *Nutrition Journal*.

[99] Santiago J. A., Potashkin J. A. (2013). Shared dysregulated pathways lead to Parkinson's disease and diabetes. *Trends in Molecular Medicine*.

[100] Kotagal V., Albin R. L., Müller M. L. T. M., Koeppe R. A., Frey K. A., Bohnen N. I. (2013). Diabetes is associated with postural instability and gait difficulty in Parkinson disease. *Parkinsonism & Related Disorders*.

[101] Palacios N., Gao X., McCullough M. L. (2011). Obesity, diabetes, and risk of Parkinson’s disease. *Movement Disorders*.

[102] Simon K. C., Chen H., Schwarzschild M., Ascherio A. (2007). Hypertension, hypercholesterolemia, diabetes, and risk of Parkinson disease. *Neurology*.

[103] Ortiz G. G., Moráles-Sánchez E. W., Pacheco-Moisés F. P. (2017). Effect of melatonin administration on cyclooxygenase-2 activity, serum levels of nitric oxide metabolites, lipoperoxides and glutathione peroxidase activity in patients with Parkinson’s disease. *Gaceta medica de Mexico*.

[104] Halliwell B. (1992). Reactive oxygen species and the central nervous system. *Journal of Neurochemistry*.

[105] Pearce R. K. B., Owen A., Daniel S., Jenner P., Marsden C. D. (1997). Alterations in the distribution of glutathione in the substantia nigra in Parkinson’s disease. *Journal of Neural Transmission*.

[106] Rahimmi A., Khosrobakhsh F., Izadpanah E., Moloudi M. R., Hassanzadeh K. (2015). N-acetylcysteine prevents rotenone-induced Parkinson's disease in rat: An investigation into the interaction of parkin and Drp1 proteins. *Brain Research Bulletin*.

[107] Wüllner U., Löschmann P.-A., Schulz J. B. (1996). Glutathione depletion potentiates MPTP and MPP+ toxicity in nigral dopaminergic neurones. *NeuroReport*.

[108] Rotruck J. T., Pope A. L., Ganther H. E., Swanson A. B., Hafeman D. G., Hoekstra W. G. (1973). Selenium: biochemical role as a component of glutathione peroxidase. *Science*.

[109] Wang H., Cheng E., Brooke S., Chang P., Sapolsky R. (2003). Over-expression of antioxidant enzymes protects cultured hippocampal and cortical neurons from necrotic insults. *Journal of Neurochemistry*.

[110] Trépanier G., Furling D., Puymirat J., Mirault M. E. (1996). Immunocytochemical localization of seleno-glutathione peroxidase in the adult mouse brain. *Neuroscience*.

[111] Kim S. H., Kim S. H., Lee J. H. (2015). Superoxide dismutase gene (SOD1, SOD2, and SOD3) polymorphisms and antituberculosis drug-induced hepatitis. *Allergy Asthma & Immunology Research*.

[112] McCord J. M., Fridovich I. (1969). Superoxide dismutase. An enzymic function for erythrocuprein (hemocuprein). *The Journal of biological chemistry*.

[113] Lindenau J., Noack H., Possel H., Asayama K., Wolf G. (2000). Cellular distribution of superoxide dismutases in the rat CNS. *Glia*.

[114] Kurobe N., Suzuki F., Okajima K., Kato K. (1990). Sensitive enzyme immunoassay for human Cu/Zn superoxide dismutase. *Clinica Chimica Acta*.

[115] Milani P., Ambrosi G., Gammoh O., Blandini F., Cereda C. (2013). SOD1 and DJ-1 converge at Nrf2 pathway: a clue for antioxidant therapeutic potential in neurodegeneration. *Oxidative Medicine and Cellular Longevity*.

[116] Choi J., Rees H. D., Weintraub S. T., Levey A. I., Chin L. S., Li L. (2005). Oxidative modifications and aggregation of Cu,Zn-superoxide dismutase associated with Alzheimer and Parkinson diseases. *Journal of Biological Chemistry*.

[117] Belluzzi E., Bisaglia M., Lazzarini E., Tabares L. C., Beltramini M., Bubacco L. (2012). Human SOD2 modification by dopamine quinones affects enzymatic activity by promoting its aggregation: possible implications for Parkinson’s disease. *PLoS One*.

[118] Hardeland R., Poeggeler B. (2003). Non-vertebrate melatonin. *J Pineal Res.*.

[119] Sae-Teaw M., Johns J., Johns N. P., Subongkot S. (2013). Serum melatonin levels and antioxidant capacities after consumption of pineapple, orange, or banana by healthy male volunteers. *Journal of Pineal Research*.

[120] Tan D. X., Zanghi B. M., Manchester L. C., Reiter R. J. (2014). Melatonin identified in meats and other food stuffs: potentially nutritional impact. *Journal of Pineal Research*.

[121] Aguilera Y., Herrera T., Benítez V. (2015). Estimation of scavenging capacity of melatonin and other antioxidants: contribution and evaluation in germinated seeds. *Food Chemistry*.

[122] Meng X., Li Y., Li S. (2017). Dietary sources and bioactivities of melatonin. *Nutrients*.

[123] Carrillo-Vico A., Reiter R. J., Lardone P. J. (2006). The modulatory role of melatonin on immune responsiveness. *Current Opinion in Investigational Drugs*.

[124] Li F., Li S., Li H. B. (2013). Antiproliferative activity of peels, pulps and seeds of 61 fruits. *Journal of Functional Foods*.

[125] Sewerynek E. (2002). Melatonin and the cardiovascular system. *Neuroendocrinology Letters*.

[126] Sofic E., Rimpapa Z., Kundurovic Z. (2005). Antioxidant capacity of the neurohormone melatonin. *Journal of Neural Transmission*.

[127] Srinivasan V., Pandi-Perumal S. R., Cardinali D. P., Poeggeler B., Hardeland R. (2006). Melatonin in Alzheimer’s disease and other neurodegenerative disorders. *Behavioral and Brain Functions*.

[128] Sandyk R. (2009). Pineal melatonin functions: possible relevance to Parkinson’s disease. *International Journal of Neuroscience*.

[129] Adi N., Mash D. C., Ali Y., Singer C., Shehadeh L., Papapetropoulos S. (2010). Melatonin MT_1_ and MT_2_ receptor expression in Parkinson’s disease. *Medical Science Monitor*.

[130] Reiter R. J., Tan D. X., Galano A. (2014). Melatonin: exceeding expectations. *Physiology*.

[131] Rodriguez C., Mayo J. C., Sainz R. M. (2004). Regulation of antioxidant enzymes: a significant role for melatonin. *Journal of Pineal Research*.

[132] Tapias V., Escames G., López L. C. (2009). Melatonin and its brain metabolite N^1^-acetyl-5-methoxykynuramine prevent mitochondrial nitric oxide synthase induction in parkinsonian mice. *Journal of Neuroscience Research*.

[133] Mayo J. C., Sainz R. M., Uria H., Antolin I., Esteban M. M., Rodriguez C. (1998). Melatonin prevents apoptosis induced by 6-hydroxydopamine in neuronal cells: implications for Parkinson’s disease. *Journal of Pineal Research*.

[134] Jin B. K., Shin D. Y., Jeong M. Y. (1998). Melatonin protects nigral dopaminergic neurons from 1-methyl-4-phenylpyridinium (MPP^+^) neurotoxicity in rats. *Neuroscience Letters*.

[135] Bassani T. B., Gradowski R. W., Zaminelli T. (2014). Neuroprotective and antidepressant-like effects of melatonin in a rotenone- induced Parkinson's disease model in rats. *Brain Research*.

[136] Dowling G. A., Mastick J., Colling E., Carter J. H., Singer C. M., Aminoff M. J. (2005). Melatonin for sleep disturbances in Parkinson's disease. *Sleep Medicine*.

[137] Srinivasan V., De Berardis D., Partonen T., Zakaria R., Othman Z. (2014). The use of melatonin for treating sleep disorders in patients with Parkinson’s disease. *ChronoPhysiology and Therapy*.

[138] Medeiros C. A. M., de Bruin P. F. C., Lopes L. A., Magalhães M. C., de Lourdes Seabra M., de Bruin V. M. S. (2007). Effect of exogenous melatonin on sleep and motor dysfunction in Parkinson’s disease. *Journal of Neurology*.

[139] Medina-Navarro R., Duran-Reyes G., Hicks J. J. (2009). Pro-oxidating properties of melatonin in the in vitro interaction with the singlet oxygen. *Endocrine Research*.

[140] Buyukavci M., Ozdemir O., Buck S., Stout M., Ravindranath Y., Savasan S. (2006). Melatonin cytotoxicity in human leukemia cells: relation with its pro-oxidant effect. *Fundamental and Clinical Pharmacology*.

[141] Clapp-Lilly K. L., Smith M. A., Perry G., Harris P. L., Zhu X., Duffy L. K. (2001). Melatonin acts as antioxidant and pro-oxidant in an organotypic slice culture model of Alzheimer’s disease. *Neuroreport*.

[142] Radogna F., Paternoster L., De Nicola M. (2009). Rapid and transient stimulation of intracellular reactive oxygen species by melatonin in normal and tumor leukocytes. *Toxicology and Applied Pharmacology*.

[143] Sánchez-Hernández D., Anderson G. H., Poon A. N. (2016). Maternal fat-soluble vitamins, brain development, and regulation of feeding behavior: an overview of research. *Nutrition Research*.

[144] Chawla J., Kvarnberg D. (2014). Hydrosoluble vitamins. *Neurologic Aspects of Systemic Disease Part II*.

[145] Zhao X., Zhang M., Li C., Jiang X., Su Y., Zhang Y. (2019). Benefits of vitamins in the treatment of Parkinson’s disease. *Oxidative Medicine and Cellular Longevity*.

[146] Sauberlich H. E. (1984). Implications of nutritional status on human biochemistry, physiology, and health. *Clinical Biochemistry*.

[147] Mikkelsen K., Stojanovska L., Tangalakis K., Bosevski M., Apostolopoulos V. (2016). Cognitive decline: a vitamin B perspective. *Maturitas*.

[148] Fukushima T. (2005). Niacin metabolism and Parkinson’s disease. *Environmental Health and Preventive Medicine*.

[149] Aoyama K., Matsubara K., Kondo M. (2001). Nicotinamide- *N* -methyltransferase is higher in the lumbar cerebrospinal fluid of patients with Parkinson's disease. *Neuroscience Letters*.

[150] Griffin S. M., Pickard M. R., Orme R. P., Hawkins C. P., Fricker R. A. (2013). Nicotinamide promotes neuronal differentiation of mouse embryonic stem cells *in vitro*. *NeuroReport*.

[151] Oudemans-van Straaten H. M., Spoelstra-de Man A. M., de Waard M. C. (2014). Vitamin C revisited. *Critical Care*.

[152] Grosso G., Bei R., Mistretta A. (2013). Effects of vitamin C on health: a review of evidence. *Frontiers in Bioscience*.

[153] Smirnoff N. (2018). Ascorbic acid metabolism and functions: a comparison of plants and mammals. *Free Radical Biology and Medicine*.

[154] Jaiswal S. K., Gupta V. K., Ansari M. D., Siddiqi N. J., Sharma B. (2017). Vitamin C acts as a hepatoprotectant in carbofuran treated rat liver slices *in vitro*. *Toxicology Reports*.

[155] Peterson A. L., Mancini M., Horak F. B. (2013). The relationship between balance control and vitamin D in Parkinson’s disease–a pilot study. *Movement Disorders*.

[156] Eyles D. W., Smith S., Kinobe R., Hewison M., McGrath J. J. (2005). Distribution of the Vitamin D receptor and 1*α*-hydroxylase in human brain. *Journal of Chemical Neuroanatomy*.

[157] Sleeman I., Aspray T., Lawson R. (2017). The role of vitamin D in disease progression in early Parkinson’s disease. *Journal of Parkinson's Disease*.

[158] Zhou Z., Zhou R., Zhang Z., Li K. (2019). The association between vitamin D status, vitamin D supplementation, sunlight exposure, and Parkinson’s disease: a systematic review and meta-analysis. *Medical Science Monitor*.

[159] Graf S., Egert S., Heer M. (2011). Effects of whey protein supplements on metabolism: evidence from human intervention studies. *Current Opinion in Clinical Nutrition and Metabolic Care*.

[160] Svanborg S., Johansen A. G., Abrahamsen R. K., Skeie S. B. (2015). The composition and functional properties of whey protein concentrates produced from buttermilk are comparable with those of whey protein concentrates produced from skimmed milk. *Journal of Dairy Science*.

[161] Haug A., Høstmark A. T., Harstad O. M. (2007). Bovine milk in human nutrition–a review. *Lipids in Health and Disease*.

[162] Park Y. W., Nam M. S. (2015). Bioactive peptides in milk and dairy products: a review. *Korean Journal for Food Science of Animal Resources*.

[163] Ebaid H., Salem A., Sayed A., Metwalli A. (2011). Whey protein enhances normal inflammatory responses during cutaneous wound healing in diabetic rats. *Lipids in Health and Disease*.

[164] Kume H., Okazaki K., Sasaki H. (2014). Hepatoprotective effects of whey protein on D-galactosamine-induced hepatitis and liver fibrosis in rats. *Bioscience, biotechnology, and biochemistry*.

[165] Athira S., Mann B., Sharma R., Kumar R. (2013). Ameliorative potential of whey protein hydrolysate against paracetamol-induced oxidative stress. *Journal of Dairy Science*.

[166] Flaim C., Kob M., Di Pierro A. M., Herrmann M., Lucchin L. (2017). Effects of a whey protein supplementation on oxidative stress, body composition and glucose metabolism among overweight people affected by diabetes mellitus or impaired fasting glucose: a pilot study. *The Journal of Nutritional Biochemistry*.

[167] Reyes R. C., Cittolin-Santos G. F., Kim J. E. (2016). Neuronal glutathione content and antioxidant capacity can be normalized in situ by N-acetyl cysteine concentrations attained in human cerebrospinal fluid. *Neurotherapeutics*.

[168] Katz M., Won S. J., Park Y. (2015). Cerebrospinal fluid concentrations of N-acetylcysteine after oral administration in Parkinson's disease. *Parkinsonism & Related Disorders*.

[169] Sheard J. M., Ash S., Mellick G. D., Silburn P. A., Kerr G. K. (2013). Malnutrition in a sample of community-dwelling people with Parkinson’s disease. *PloS ONE*.

[170] Sheard J. M., Ash S., Silburn P. A., Kerr G. K. (2011). Prevalence of malnutrition in Parkinson’s disease: a systematic review. *Nutrition Reviews*.

[171] Cano-de-la-Cuerda R., Pérez-de-Heredia M., Miangolarra-Page J. C., Muñoz-Hellín E., Fernández-de-las-Peñas C. (2010). Is there muscular weakness in Parkinson’s disease?. *American Journal of Physical Medicine & Rehabilitation*.

[172] Tosukhowong P., Boonla C., Dissayabutra T. (2016). Biochemical and clinical effects of Whey protein supplementation in Parkinson's disease: A pilot study. *Journal of the Neurological Sciences*.

[173] Wang L., Xiong N., Huang J. (2017). Protein-restricted diets for ameliorating motor fluctuations in Parkinson’s disease. *Frontiers in Aging Neuroscience*.

[174] Borchers A. T., Keen C. L., Hannum S. M., Gershwin M. E. (2000). Cocoa and chocolate: composition, bioavailability, and health implications. *Journal of Medicinal Food*.

[175] Carlsen M. H., Halvorsen B. L., Holte K. (2010). The total antioxidant content of more than 3100 foods, beverages, spices, herbs and supplements used worldwide. *Nutrition Journal*.

[176] Williams R. J., Spencer J. P., Rice-Evans C. (2004). Flavonoids: antioxidants or signalling molecules?. *Free Radical Biology and Medicine*.

[177] Heim K. E., Tagliaferro A. R., Bobilya D. J. (2002). Flavonoid antioxidants: chemistry, metabolism and structure-activity relationships. *The Journal of Nutritional Biochemistry*.

[178] Ferrali M., Signorini C., Caciotti B. (1997). Protection against oxidative damage of erythrocyte membrane by the flavonoid quercetin and its relation to iron chelating activity. *FEBS Letters*.

[179] Latif R., Alsunni A. A. (2016). Effects of chocolate intake on oxidative stress/oxidant-antioxidant balance in medical students: a controlled clinical trial. *Saudi Journal of Medicine and Medical Sciences*.

[180] Cooper K. A., Campos-Giménez E., Jiménez-Alvarez D., Nagy K., Donovan J. L., Williamson G. (2007). Rapid reversed Phase Ultra-Performance liquid chromatography analysis of the Major cocoa polyphenols and inter-relationships of their concentrations in chocolate. *Journal of Agricultural and Food Chemistry*.

[181] Vlachopoulos C., Alexopoulos N., Stefanadis C. (2006). Effect of dark chocolate on arterial function in healthy individuals: cocoa instead of ambrosia?. *Current Hypertension Reports*.

[182] Grassi D., Lippi C., Necozione S., Desideri G., Ferri C. (2005). Short-term administration of dark chocolate is followed by a significant increase in insulin sensitivity and a decrease in blood pressure in healthy persons. *The American Journal of Clinical Nutrition*.

[183] Maskarinec G. (2009). Cancer protective properties of cocoa: a review of the epidemiologic evidence. *Nutrition and Cancer*.

[184] Khawaja O., Gaziano J. M., Djoussé L. (2011). Chocolate and coronary heart disease: a systematic review. *urrent Atherosclerosis Reports*.

[185] Ramiro-Puig E., Casadesús G., Lee H. G. (2009). Neuroprotective effect of cocoa flavonoids on in vitro oxidative stress. *European Journal of Nutrition*.

[186] Wolz M., Kaminsky A., Löhle M., Koch R., Storch A., Reichmann H. (2009). Chocolate consumption is increased in Parkinson’s disease. Results from a self-questionnaire study. *Journal of Neurology*.

[187] Wolz M., Schleiffer C., Klingelhöfer L. (2012). Comparison of chocolate to cacao-free white chocolate in Parkinson’s disease: a single-dose, investigator-blinded, placebo-controlled, crossover trial. *Journal of Neurology*.

[188] Irsfeld M., Spadafore M., Prüß B. M. (2013). *β*-Phenylethylamine, a small molecule with a large impact. *Webmedcentral*.

[189] Pastore P., Favaro G., Badocco D., Tapparo A., Cavalli S., Saccani G. (2005). Determination of biogenic amines in chocolate by ion chromatographic separation and pulsed integrated amperometric detection with implemented wave- form at Au disposable electrode. *Journal of Chromatography A*.

[190] Berry M. D. (2004). Mammalian central nervous system trace amines. Pharmacologic amphetamines, physiologic neuromodulators. *Journal of Neurochemistry*.

[191] Sengupta T., Mohanakumar K. P. (2010). 2-Phenylethylamine, a constituent of chocolate and wine, causes mitochondrial complex-I inhibition, generation of hydroxyl radicals and depletion of striatal biogenic amines leading to psycho-motor dysfunctions in Balb/c mice. *Neurochemistry International*.

[192] Ortmann R., Schaub M., Felner A., Lauber J., Christen P., Waldmeier P. C. (1984). Phenylethylamine-induced stereotypies in the rat: a behavioral test system for assessment of MAO-B inhibitors. *Psychopharmacology*.

[193] Lapin I. P. (1996). Antagonism by CPP, (±)-3-(2-carboxypiperazin-4-yl)-propyl-1-phosphonic acid, of *β*-phenylethylamine (PEA)-induced hypermotility in mice of different strains. *Pharmacology Biochemistry and Behavior*.

[194] Borah A., Paul R., Mazumder M. K., Bhattacharjee N. (2013). Contribution of *β*-phenethylamine, a component of chocolate and wine, to dopaminergic neurodegeneration: implications for the pathogenesis of Parkinson’s disease. *Neuroscience bulletin*.

[195] Goedert M. (2001). Alpha-synuclein and neurodegenerative diseases. *Nature Reviews Neuroscience*.

[196] Oueslati A. (2016). Implication of alpha-synuclein phosphorylation at S129 in synucleinopathies: what have we learned in the last decade?. *Journal of Parkinson's disease*.

[197] Fujiwara H., Hasegawa M., Dohmae N. (2002). *α*-Synuclein is phosphorylated in synucleinopathy lesions. *Nature cell biology*.

[198] Wu J., Tolstykh T., Lee J., Boyd K., Stock J. B., Broach J. R. (2000). Carboxyl methylation of the phosphoprotein phosphatase 2A catalytic subunit promotes its functional association with regulatory subunits in vivo. *The EMBO journal*.

[199] Park H. J., Lee K. W., Park E. S. (2016). Dysregulation of protein phosphatase 2A in Parkinson disease and dementia with Lewy bodies. *Annals of clinical and translational neurology*.

[200] Martini D., del Bo’ C., Tassotti M. (2016). Coffee consumption and oxidative stress: a review of human intervention studies. *Molecules*.

[201] Luan Y., Ren X., Zheng W. (2018). Chronic caffeine treatment protects against *α*-synucleinopathy by reestablishing autophagy activity in the mouse striatum. *Frontiers in neuroscience*.

[202] Lee K. W., Im J. Y., Woo J. M. (2013). Neuroprotective and anti-inflammatory properties of a coffee component in the MPTP model of Parkinson’s disease. *Neurotherapeutics*.

[203] Yan R., Zhang J., Park H. J. (2018). Synergistic neuroprotection by coffee components eicosanoyl-5-hydroxytryptamide and caffeine in models of Parkinson’s disease and DLB. *Proceedings of the National Academy of Sciences*.

[204] Trinh K., Andrews L., Krause J. (2010). Decaffeinated coffee and nicotine-free tobacco provide neuroprotection in Drosophila models of Parkinson’s disease through an NRF2-dependent mechanism. *Journal of Neuroscience*.

[205] Jia N., Han K., Kong J. J. (2013). (−)-Epigallocatechin-3-gallate alleviates spatial memory impairment in APP/PS1 mice by restoring IRS-1 signaling defects in the hippocampus. *Molecular and cellular biochemistry*.

[206] Kelemen W. L., Creeley C. E. (2003). State-dependent memory effects using caffeine and placebo do not extend to metamemory. *The Journal of general psychology*.

[207] Arnold M. E., Petros T. V., Beckwith B. E., Coons G., Gorman N. (1987). The effects of caffeine, impulsivity, and sex on memory for word lists. *Physiology & behavior*.

[208] Institute of Medicine Committee on Military Nutrition Research (2002). Caffeine for the sustainment of mental task performance: formulations for military operations. *Nutrition Today*.

[209] Corley J., Jia X., Kyle J. A. M. (2010). Caffeine consumption and cognitive function at age 70: the Lothian Birth Cohort 1936 study. *Psychosomatic medicine*.

[210] Yeung A. W. K., Tzvetkov N. T., El-Tawil O. S., Bungǎu S. G., Abdel-Daim M. M., Atanasov A. G. (2019). Antioxidants: scientific literature landscape analysis. *Oxidative medicine and cellular longevity*.

[211] Uddin M. S., Hossain M. F., Mamun A. A. (2020). Exploring the multimodal role of phytochemicals in the modulation of cellular signaling pathways to combat age-related neurodegeneration. *Science of The Total Environment*.

[212] Abushouk A. I., Negida A., Ahmed H., Abdel-Daim M. M. (2017). Neuroprotective mechanisms of plant extracts against MPTP induced neurotoxicity: Future applications in Parkinson's disease. *Biomedicine & Pharmacotherapy*.

[213] Rahman M. A., Rahman M. R., Zaman T. (2020). Emerging potential of naturally occurring autophagy modulators against neurodegeneration. *Current Pharmaceutical Design*.

[214] Kumar A., Ekavali, Chopra K., Mukherjee M., Pottabathini R., Dhull D. K. (2015). Current knowledge and pharmacological profile of berberine: an update. *European Journal of Pharmacology*.

[215] Jiang X. W., Zhang Y., Zhu Y. L. (2013). Effects of berberine gelatin on recurrent aphthous stomatitis: a randomized, placebo-controlled, double-blind trial in a Chinese cohort. *Oral Surgery, Oral Medicine, Oral Pathology and Oral Radiology*.

[216] Li H. L., Han T., Liu R. H., Zhang C., Chen H. S., Zhang W. D. (2008). Alkaloids from Corydalis saxicola and their anti-hepatitis B virus activity. *Chemistry & Biodiversity*.

[217] Wang H., Zhu C., Ying Y., Luo L., Huang D., Luo Z. (2018). Metformin and berberine, two versatile drugs in treatment of common metabolic diseases. *Oncotarget*.

[218] Ji H. F., Shen L. (2011). Berberine: a potential multipotent natural product to combat Alzheimer’s disease. *Molecules*.

[219] Durairajan S. S. K., Liu L.-F., Lu J.-H. (2012). Berberine ameliorates *β*-amyloid pathology, gliosis, and cognitive impairment in an Alzheimer's disease transgenic mouse model. *Neurobiology of Aging*.

[220] Kim M., Cho K.-H., Shin M.-S. (2014). Berberine prevents nigrostriatal dopaminergic neuronal loss and suppresses hippocampal apoptosis in mice with Parkinson’s disease. *International Journal of Molecular Medicine*.

[221] Kwon I. H., Choi H. S., Shin K. S. (2010). Effects of berberine on 6-hydroxydopamine-induced neurotoxicity in PC12 cells and a rat model of Parkinson's disease. *Neuroscience Letters*.

[222] Shin K. S., Choi H. S., Zhao T. T. (2013). Neurotoxic effects of berberine on long-term L-DOPA administration in 6-hydroxydopamine-lesioned rat model of Parkinson’s disease. *Archives of Pharmacal Research*.

[223] Hewlings S., Kalman D. (2017). Curcumin: a review of its’ effects on human health. *Foods*.

[224] Zbarsky V., Datla K. P., Parkar S., Rai D. K., Aruoma O. I., Dexter D. T. (2009). Neuroprotective properties of the natural phenolic antioxidants curcumin and naringenin but not quercetin and fisetin in a 6-OHDA model of Parkinson’s disease. *Free Radical Research*.

[225] Nam S. M., Choi J. H., Yoo D. Y. (2014). Effects of curcumin (Curcuma longa) on learning and spatial memory as well as cell proliferation and neuroblast differentiation in adult and aged mice by upregulating brain-derived neurotrophic factor and CREB signaling. *Journal of Medicinal Food*.

[226] Liu D., Wang Z., Gao Z. (2014). Effects of curcumin on learning and memory deficits, BDNF, and ERK protein expression in rats exposed to chronic unpredictable stress. *Behavioural Brain Research*.

[227] Nguyen T. T., Vuu M. D., Huynh M. A., Yamaguchi M., Tran L. T., Dang T. P. T. (2018). Curcumin effectively rescued Parkinson’s disease-like phenotypes in a novel Drosophila melanogaster model with dUCH knockdown. *Oxidative Medicine and Cellular Longevity*.

[228] Li Y., Yao J., Han C. (2016). Quercetin, inflammation, and immunity. *Nutrients*.

[229] Stewart L. K., Soileau J. L., Ribnicky D. (2008). Quercetin transiently increases energy expenditure but persistently decreases circulating markers of inflammation in C57BL/6J mice fed a high-fat diet. *Metabolism*.

[230] Dong Y. S., Wang J. L., Feng D. Y. (2014). Protective effect of quercetin against oxidative stress and brain edema in an experimental rat model of subarachnoid hemorrhage. *International Journal of Medical Sciences*.

[231] Singh S., Jamwal S., Kumar P. (2017). Neuroprotective potential of quercetin in combination with piperine against 1-methyl-4-phenyl-1,2,3,6-tetrahydropyridine-induced neurotoxicity. *Neural Regeneration Research*.

